# The Red Cell Volume in a Hospital Population

**Published:** 1972-07

**Authors:** C. D. R. Pengelly, Vivien McKenzie

**Affiliations:** Consultant Physician; Research Assistant from The Grange Hospital, Weaverham, Cheshire


### The Red Cell Volume in a Hospital Population by

C. D. R. Pengelly, M.D. (Brist.), F.R.C.P., F.R.C.P.E.,
Consultant Physician, and

Vivien McKenzie, B.Sc. (Glas.)
Research Assistant from The Grange Hospital, Weaverham, Cheshire

### INTRODUCTION

The normal values for red cell volume have been widely accepted as 26 to 33 ml/kg in males and 22 to 29 ml/kg in females (Dacie and Lewis 1966) and Mollison (1967) quotes figures of about 30 ml/kg in males and 23.4 to 27 ml/kg in females, referring to five papers reporting investigations with radioactive iron ($^{55}$Fe and $^{59}$Fe) or radioactive phosphorous ($^{32}$P). Using radioactive chromium ($^{51}$Cr), Retzlaff, Tauxe, Kiely and Stroebel (1969) found values which give approximately the following: in 40 "normal" males 26.8 ml/kg; in 38 "normal" females 23.2 ml/kg; and in 12 "obese" females 17.3 ml/kg. The mean weight of the latter was 86.2 kg (189.6 lb.).

The present study was undertaken to determine the red cell volume in the normal subject using $^{51}$Cr. In view of the difficulties in obtaining large numbers of healthy volunteers, some patients who were willing to volunteer for the test were investigated. These were all patients who were about to be discharged from hospital and had been up and about in the wards for at least seven days, and who had no clinical evidence of any heart failure, bleeding or abnormality of fluid balance or active infection.

### METHOD

The subjects were weighed without any clothing and were then asked to lie on a bed for the duration of the estimation. This was carried out essentially by the method of Mollison and Veall (1955) using 30 to 50 microcuries of $^{51}$Cr. About 25 ml of radioactive red cell suspension was prepared and exactly 20 ml injected into one of the subject's antecubital veins. From the remainder of the suspension two 1 in 50 standards were made up in 50 ml flasks using as diluent 2% sodium chromate solution. A few particles of saponin were added to complete haemolysis. A 1 in 50 dilution of the supernatant from the injection sample was also made. Two 15 ml specimens of blood were taken from the arm of the subject opposite to that used for the injection 25 and 30 minutes later. Heparin was used as anticoagulant. Packed cell volumes were estimated by the method of Chaplin and Mollison (1952) in duplicate, centrifuging being carried out for 55 minutes. The results were then corrected for trapped plasma. The two blood specimens were haemolysed by the addition to each of approximately 30 mg of saponin. Radioactive assay was carried out by means of a 10 ml annular sample cup and Isotope Developments Ltd. apparatus (crystal, lead shielding type 722, scintillation counter type 663 and scaler type 1700). All counts were expressed as counts per 100 seconds minus background.

From the mean of the counts of the two 1 in 50 injection standards was subtracted the count of the supernatant standard adjusted for the haematocrit of the red cell suspension and the result divided by the mean of the counts of the two blood specimens after both had been expressed in terms of 100% red cells. This gave the result of the red cell volume directly in litres which was then expressed as ml/kg body weight.

### ACCURACY

The two 20 ml syringes (B and C) employed for injecting the red cell suspension were of glass and metal nozzle construction. They were recalibrated by making a diamond scratch on the piston and another on the barrel exactly opposite after 20 ml of distilled water had been sucked into the syringe from a watch glass with a Gillette No. 1 disposable needle fitted, the "dead space" having first been filled with distilled water. The calibrations were checked by weighing the delivered distilled water ten times, using a different Gillette No. 1 needle each time. The two ml pipettes (6 and 9) for making the 1 in 50 injection standards and the two 10 ml pipettes (S and T) used for filling the counting cup with the radioactive specimens were checked in the same way. The two 50 ml flasks (A and Z) were checked by weighing their content of distilled water after filling each flask ten times.

The following results were obtained :

| | Inaccuracy of Means per cent | Standard Deviations per cent |
|---|---|---|
| Syringe B | 0.50 | 0.10 |
| Syringe C | 0.02 | 0.10 |
| Pipette 6 | 0.08 | 0.10 |
| Pipette 9 | 0.08 | 0.13 |
| Pipette S | 0.31 | 0.04 |
| Pipette T | 0.21 | 0.02 |
| Flask A | 0.12 | 0.02 |
| Flask Z | 0.06 | 0.03 |

The haematocrit tubes were of British Standard (B.S.) 2554 and were read to one tenth of a division.

The mean background count was 1.3 counts per second; that of the red cell standards was 48 c.p.s. and of the blood specimens 21 c.p.s. The radioactivity of the supernatant of the injected standards was always less than 1% of the corresponding red cell standard.

It is concluded that the total systematic error is unlikely to exceed 1% and that the random error due to counting is about the same magnitude.

### RESULTS

The results of the red cell volume estimate together with packed cell volume, weight, age and description of the subjects tested are given in Tables 1 to 7. Table 7 is a summary of all the other tables.

### TABLE 1

**Normal Volunteers — Males**

| Case No. | Occupation | Age (yrs) | Weight (kg) | P.C.V. % | R.C.V. ml/kg |
|---|---|---|---|---|---|
| 1 | Clerk ............ | 42 | 82.0 | 47.7 | 29.9 |
| 1 | Clerk ............ | 21 | 68.7 | 39.9 | 25.1 |
| 3 | Nurse ......... | 48 | 90.3 | 46.9 | 24.8 |
| 4 | Hospital Secretary ... | 53 | 75.5 | 47.3 | 29.6 |
| 5 | Electrician ...... | 43 | 76.9 | 43.2 | 22.6 |
| | MEAN | | | 45.0 | 26.4 |
| | STANDARD DEVIATION | | | 3.0 | 2.9 |

### TABLE 2

**Normal Volunteers — Females**

| Case No. | Occupation | Age (yrs) | Weight (kg) | P.C.V. % | R.C.V. ml/kg |
|---|---|---|---|---|---|
| 1 | Secretary ...... | 48 | 61.4 | 36.9 | 24.1 |
| 2 | Nurse ......... | 48 | 51.8 | 38.9 | 21.0 |
| 3 | Nurse ......... | 51 | 76.6 | 41.1 | 20.4 |
| 4 | Nurse ......... | 47 | 94.0 | 44.4 | 23.6 |
| 5 | Cleaner ......... | 55 | 65.0 | 32.5 | 21.4 |
| 6 | Nurse ......... | 55 | 52.7 | 35.7 | 21.2 |
| | MEAN | | | 38.3 | 22.0 |
| | STANDARD DEVIATION | | | 3.8 | 1.4 |

### TABLE 3

**Hospital Normals — Males**

| Case No. | Diagnosis | Age (yrs) | Weight (kg) | P.C.V. % | R.C.V. ml/kg |
|---|---|---|---|---|---|
| 1 | Coronary artery disease ...... | 48 | 66.1 | 44.7 | 28.0 |
| 2 | Epilepsy ......... | 43 | 69.5 | 41.0 | 26.3 |
| 3 | Controlled diabetes ...... | 80 | 72.5 | 41.4 | 20.7 |
| 4 | Coronary artery disease ...... | 48 | 63.0 | 45.3 | 27.0 |
| 5 | Coronary artery disease ...... | 53 | 82.7 | 40.4 | 17.8 |
| 6 | Coronary artery disease ...... | 62 | 58.2 | 45.1 | 27.7 |
| 7 | Coronary artery disease ...... | 53 | 86.9 | 43.7 | 24.2 |
| 8 | Cerebral arterial disease ...... | 83 | 52.7 | 42.4 | 26.1 |
| 9 | Cerebral arterial disease ...... | 60 | 70.9 | 39.6 | 21.8 |
| 10 | Pneumonia (convalescent) | 49 | 102.3 | 42.5 | 21.6 |
| 11 | Pneumonia (convalescent) | 28 | 70.8 | 39.6 | 25.5 |
| 12 | Coronary artery disease ...... | 58 | 66.0 | 40.8 | 25.3 |
| 13 | Coronary artery disease ...... | 50 | 75.2 | 41.9 | 26.2 |
| 14 | Duodenal ulcer ... | 51 | 61.8 | 38.1 | 23.2 |
| 15 | Pyrexia undiagnosed (convalescent) | 30 | 76.3 | 43.7 | 29.6 |
| 16 | Duodenal ulcer ... | 20 | 81.6 | 43.0 | 29.5 |
| 17 | Coronary artery disease ...... | 66 | 97.3 | 39.7 | 20.2 |
| | MEAN | | | 41.9 | 24.8 |
| | STANDARD DEVIATION | | | 2.1 | 3.3 |

### TABLE 4

**Hospital Normals — Females**

| Case No. | Diagnosis | Age (yrs) | Weight (kg) | P.C.V. % | R.C.V. my/kg |
|---|---|---|---|---|---|
| 1 | Gastric Ulcer ... | 46 | 51.4 | 33.9 | 22.0 |
| 2 | Gastric Ulcer ... | 56 | 54.5 | 40.0 | 24.8 |
| 3 | Gastroenteritis (convalescent) | 56 | 53.7 | 38.7 | 19.5 |
| 4 | Epilepsy ......... | 57 | 44.4 | 39.2 | 22.7 |
| 5 | Duodenal Ulcer... | 43 | 45.5 | 38.3 | 23.9 |
| | MEAN | | | 38.0 | 22.6 |
| | STANDARD DEVIATION | | | 2.1 | 1.8 |

### TABLE 5

**All Males**

| Case No. | Category | P.C.V. % | R.C.V. ml/kg |
|---|---|---|---|
| 1 | Normal volunteer ............ | 47.7 | 29.9 |
| 2 | ,, ,, ............ | 39.9 | 25.1 |
| 3 | ,, ,, ............ | 46.9 | 24.8 |
| 4 | ,, ,, ............ | 47.3 | 29.6 |
| 5 | ,, ,, ............ | 43.2 | 22.6 |
| 1 | Hospital normal* ............ | 44.7 | 28.0 |
| 2 | ,, ,, ............ | 41.0 | 26.3 |
| 3 | ,, ,, ............ | 41.4 | 20.7 |
| 4 | ,, ,, ............ | 45.3 | 27.0 |
| 5 | ,, ,, ............ | 40.4 | 17.8 |
| 6 | ,, ,, ............ | 45.1 | 27.7 |
| 7 | ,, ,, ............ | 43.7 | 24.2 |
| 8 | ,, ,, ............ | 42.4 | 26.1 |
| 9 | ,, ,, ............ | 39.6 | 21.8 |
| 10 | ,, ,, ............ | 42.5 | 21.6 |
| 11 | ,, ,, ............ | 39.6 | 25.5 |
| 12 | ,, ,, ............ | 40.8 | 25.3 |
| 13 | ,, ,, ............ | 41.9 | 26.2 |
| 14 | ,, ,, ............ | 38.1 | 23.2 |
| 15 | ,, ,, ............ | 43.7 | 29.6 |
| 16 | ,, ,, ............ | 43.0 | 29.5 |
| 17 | ,, ,, ............ | 39.7 | 20.2 |
| | MEAN | 42.6 | 25.1 |
| | STANDARD DEVIATION | 2.7 | 3.3 |

*For diagnosis see Table 3

### TABLE 6

**All Females**

| Case No. | Category | P.C.V. % | R.C.V. ml/kg |
|---|---|---|---|
| 1 | Normal volunteer ............ | 36.9 | 24.1 |
| 2 | ,, ,, ............ | 38.9 | 21.0 |
| 3 | ,, ,, ............ | 41.1 | 20.4 |
| 4 | ,, ,, ............ | 44.4 | 23.6 |
| 5 | ,, ,, ............ | 32.5 | 21.4 |
| 6 | ,, ,, ............ | 35.7 | 21.2 |
| 1 | Hospital normal* ............ | 33.9 | 22.0 |
| 2 | ,, ,, ............ | 40.0 | 24.8 |
| 3 | ,, ,, ............ | 38.7 | 19.5 |
| 4 | ,, ,, ............ | 39.2 | 22.7 |
| 5 | ,, ,, ............ | 38.3 | 23.9 |
| | MEAN | 38.2 | 22.2 |
| | STANDARD DEVIATION | 3.2 | 1.6 |

*For diagnosis see Table 4

### TABLE 7

**Summary**

| | P.C.V % Mean | P.C.V % S.D. | R.C.V. ml/kg Mean | R.C.V. ml/kg S.D. |
|---|---|---|---|---|
| Male Volunteers ............ | 45.0 | 3.0 | 26.4 | 2.9 |
| Male Hospital Normals ...... | 41.9 | 2.1 | 24.8 | 3.3 |
| All Males ...................... | 42.6 | 2.7 | 25.1 | 3.3 |
| Female Volunteers ............ | 38.3 | 3.8 | 22.0 | 1.4 |
| Female Hospital Normals ... | 38.0 | 2.1 | 22.6 | 1.8 |
| All Females .................. | 38.2 | 3.2 | 22.2 | 1.6 |
| All Volunteers ............... | 41.3 | 4.8 | 24.0 | 3.1 |
| All Hospital Normals ......... | 41.0 | 2.6 | 24.3 | 3.1 |
| All Cases ..................... | 41.1 | 3.5 | 24.2 | 3.2 |

The means and standard deviations of the red cell volume results were as follows: normal volunteers, males, mean 26.4 ml/kg (S.D. 2.9); normal volunteers, females, mean 22.0 ml/kg (S.D. 1.4); hospital "normals", males, mean 24.9 ml/kg (S.D. 3.3); hospital "normals", females, mean 22.6 ml/kg (S.D. 1.8); all males, mean 25.1 ml/kg (S.D. 3.3); all females, mean 22.2 ml/kg (S.D. 1.6).

Student t-tests revealed a difference significant at the 5% level but not at the 1% level between the results of the male volunteers and the female volunteers and between those of all the males and all the females. No significant differences were revealed in comparing any other pairs of groups.

### DISCUSSION

It will be noted that the results differ little from those accepted as normal and quoted by Dacie and Lewis (1966). The expected significant difference was found between the results of the male and female volunteers and between those of all males and all females. The reason for the lack of significance between the hospital males and the hospital females is not clear. There were of course only a relatively few hospital females and the mean red cell volume of the hospital males was rather lower than the normal volunteer males and also rather below the accepted normal range for males. Obesity did not appear to be a particular factor in lowering the red cell volume (expressed as ml/kg).

Every care has been taken in this series of estimations to keep both the systematic error and the error in radioactive counting down to a low level. Repeat tests were not done on the same subject purposely to avoid any unnecessary radioactive exposure though it would have been possible in the circumstances to check the "repeatability" of the estimation in mathematical terms.

### ACKNOWLEDGEMENTS

We are grateful to the subjects and patients who volunteered for the tests; to Professor P. R. Davis and his staff at the University of Surrey for help with the statistical calculations; to Dr. E. W. Emery of the Royal Post-graduate Medical School, Hammersmith, London, for helpful criticism; to the Manchester Regional Hospital Board for a research grant; and to Mrs. J. White for typing the manuscript.

### REFERENCES

CHAPLIN, H., jr. and MOLLISON, P. L. (1952). Correction for plasma trapped in the red cell column of the haematocrit. Blood, **7**, 1227-1238.

DACIE, J. V. and LEWIS, S. M. (1966), Practical Haematology, 3rd ed. p. 12. J. and A. Churchill, Ltd., London.

MOLLISON, P. L. (1967). Blood Transfusion in Clinical Medicine, 4th ed. pp. 134-135. Blackwell Scientific Publications, Oxford and Edinburgh.

MOLLISON, P. L. and VEALL, N. (1955). The use of the isotope $^{51}Cr$ as a label for red cells. Brit. J. Haemat, **1**, 62-74.

RETZLAFF, J. A., TAUXE, W. N., KIELY, J. M., and STROEBEL, C. F. (1969). Erythrocyte volume, plasma volume and lean body mass in adult men and women. Blood, **33**, 649-661.

